# Universal Epidemic

**Published:** 1914-03

**Authors:** 


Universal Epidemic.—Mr. Roger W. Babson says that in looking up appendicitis cases he learned that in 17 per cent of the operations for that disease the post-mortem examinations showed that the appendix was in perfect condition.

"The whole subject," he adds, "reminds me of a true story I heard in London recently. In the hospitals there, the ailment of the patient, when he is admitted, is denoted by certain letters, such as 'T. B.' for tuberculosis. An American doctor was examining these history slips when his curiosity was aroused by the number on which the letters 'G. O. K.' appeared. He said to the physician who was showing him around:

" 'There seems to be a severe epidemic of this G. O. K. in London. What is it, anyhow?'

" 'Oh, that means "God only knows," ' replied the English physician."—*Open Door.*

